# CCL17 blockade as a therapy for osteoarthritis pain and disease

**DOI:** 10.1186/s13075-018-1560-9

**Published:** 2018-04-05

**Authors:** Ming-Chin Lee, Reem Saleh, Adrian Achuthan, Andrew J. Fleetwood, Irmgard Förster, John A. Hamilton, Andrew D. Cook

**Affiliations:** 1 The University of Medicine, Department of Medicine, Royal Melbourne Hospital, Parkville, VIC 3050 Australia; 2 Immunology and Environment, Life and Medical Sciences Institute, University of Bonn, 53115 Bonn, Germany

**Keywords:** Osteoarthritis, CCL17, Inflammation, Targeting, Animal models

## Abstract

**Background:**

Granulocyte macrophage-colony stimulating factor (GM-CSF) has been implicated in the pathogenesis of a number of inflammatory diseases and in osteoarthritis (OA). We identified previously a new GM-CSF→Jmjd3→interferon regulatory factor 4 (IRF4)→chemokine (c-c motif) ligand 17 (CCL17) pathway, which is important for the development of inflammatory arthritis pain and disease. Tumour necrosis factor (TNF) can also be linked with this pathway. Here we investigated the involvement of the pathway in OA pain and disease development using the GM-CSF-dependent collagenase-induced OA (CiOA) model.

**Methods:**

CiOA was induced in C57BL/6 wild-type (WT), *Irf4*^*−/−*^, *Ccl17*^*E/E*^, *Ccr4*^*−/−*^, *Tnf*^*−/−*^ and *GM-CSF*^*−/−*^ mice. Additionally, therapeutic targeting of CCL17, Jmjd3 and cyclooxygenase 2 (COX-2) was evaluated. Development of pain (assessment of weight distribution) and OA disease (histologic scoring of synovitis, cartilage destruction and osteophyte size) were assessed. Synovial joint cells, including neutrophils, macrophages, fibroblasts and endothelial cells, were isolated (cell sorting) and gene expression analyzed (quantitative PCR).

**Results:**

Studies in the gene-deficient mice indicated that IRF4, CCL17 and the CCL17 receptor, CCR4, but not TNF, were required for CiOA pain and optimal cartilage destruction and osteophyte size. Therapeutic neutralization of CCL17 and Jmjd3 ameliorated both pain and disease, whereas the COX-2 inhibitor only ameliorated pain. In the synovium *Ccl17* mRNA was expressed only in the macrophages in a GM-CSF-dependent and IRF4-dependent manner.

**Conclusions:**

The GM-CSF→Jmjd3→IRF4→CCL17 pathway is important for the development of CiOA, with CCL17 thus being a potential therapeutic target for the treatment of both OA pain and disease.

**Electronic supplementary material:**

The online version of this article (10.1186/s13075-018-1560-9) contains supplementary material, which is available to authorized users.

## Background

Osteoarthritis (OA) is the most common musculoskeletal disorder, characterized by chronic joint pain and substantial functional impairment [1]. Although OA has historically not been considered an inflammatory condition, a growing body of evidence supports the involvement of synovial inflammation in the observed cartilage degradation and bone erosion [2–6]. Proinflammatory cytokines are likely to be critical in driving such inflammation.

The collagenase-induced OA (CiOA) model involves the induction of joint instability by intra-articular injection of collagenase [7, 8] leading to joint damage, including cartilage matrix breakdown [9, 10], macrophage-mediated osteophyte formation [11, 12], as well as pain [8, 13], thereby mimicking features of the human disease [14]. We have previously demonstrated that granulocyte macrophage-colony stimulating factor (GM-CSF) is a key mediator in the CiOA model [8]. Both prophylactic, and notably therapeutic, blockade of GM-CSF using a neutralizing monoclonal antibody (mAb) have been shown to be effective at ameliorating CiOA-induced pain and disease [8]. As a result there is a current phase II trial in hand OA using this approach [15].

In addition to OA, GM-CSF has been implicated in the development of inflammatory pain and arthritic pain and disease [16–19], and blockade of GM-CSF and its receptor are currently showing promise in rheumatoid arthritis (RA) trials [15]. Regarding the mode of action of GM-CSF, we have recently reported that GM-CSF induces the chemokine, chemokine (c-c motif) ligand 17 (CCL17), via Jmjd3-regulated interferon regulatory factor 4 (IRF4), to mediate inflammation, and that blockade of CCL17 can ameliorate GM-CSF-dependent inflammatory pain and arthritic pain and disease [18]. Furthermore, models in which tumour necrosis factor (TNF) is necessary can utilize this pathway [20].

In the current study we provide evidence that the GM-CSF→Jmjd3→IRF4→CCL17 pathway, originally identified in human and murine monocytes/macrophages [18], is required for CiOA pain and optimal arthritis development; however TNF is not involved.

## Methods

### Mice

The following were used: *Tnf*^*−/−*^ [21], *Ccr4*^*−/−*^ [22], *GM-CSF*^*−/−*^ (from Ludwig Institute for Cancer Research) [23], *Irf4*^−/−^ (from TW Mak) [24] and *Ccl17* gene-deficient (*Ccl17*^*E/E*^) mice (in which both copies of *Ccl17* have been replaced by enhanced green fluorescent protein (EGFP)) [25], all backcrossed onto the C57BL/6 background (from the Walter and Eliza Hall Institute). Mice were fed standard rodent chow and water ad libitum. Mice of both sexes (8–12 weeks) were used; experiments were approved by The University of Melbourne Animal Ethics Committee.

### CiOA

CiOA was induced as published [8, 26]. Briefly, mice received an intra-articular injection of one unit of collagenase type VII (Sigma-Aldrich) on days 0 and 2 to induce joint instability. At various time points, knee joints were collected for histology or cell isolation.

### Pain reading

As a validated indicator of arthritic knee pain, the differential distribution of weight between the inflamed limb relative to the non-inflamed limb was measured using an incapacitance meter (IITC Life Science Inc, USA) [8, 17, 18, 27, 28]. Three measurements were taken for each time point and averaged.

### Therapeutic treatment

Mice with CiOA were treated therapeutically, beginning once pain was evident (days 20–23), with (i) anti-mouse CCL17 mAb (150 μg intraperitoneal (i.p.), clone 110,904, R&D Systems) or isotype control mAb (GL117.41, Schering BioPharma) given twice weekly, (ii) the Jmjd3 inhibitor, GSK-J4 (0.5 mg/kg i.p., Santa Cruz Biotechnology), or vehicle (dimethyl sulfoxide (DMSO)) given daily for 5 days followed by twice weekly or (iii) the cyclooxygenase 2 (COX-2) inhibitor, SC58125 (1 mg/kg i.p., Tocris), or vehicle (DMSO) given weekly.

### Histologic assessment

At termination, the knee joints were removed, fixed, decalcified and paraffin embedded as described previously [8, 18, 28]. Week-2 frontal sections (7 μm) were cut at various depths, stained with H&E and scored for synovitis from 0 (normal) to 3 (severe), as described before [8]. As previously published [8, 26, 29], week-6 sections were cut at various depths, stained with safranin O and fast green and scored for cartilage damage in terms of the OA depth into cartilage (grade) from 0 (normal) to 6 (bone loss, remodelling, deformation), and amount of cartilage affected (stage), from 0 (< 10% involvement) to 5 (> 75% involvement), in the lateral tibia (LT), lateral femur (LF), medial tibia (MT) and medial femur (MF) — the grade and stage values were multiplied to give an OA score. Three sections per knee joint at different depths were scored and the average OA score per joint region was calculated. This scoring is a more detailed version of the Osteoarthritis Research Society International (OARSI) scoring system for the mouse [30]. Finally, for each mouse, the OA scores from the LT, LF, MT and MF were also averaged to give a mean histologic score. Osteophyte size was assessed using ImageJ software (National Institutes of Health, Bethesda, MD, USA) [8].

### Cell sorting

Joint tissues were harvested from CiOA mice and digested with 1 mg/kg collagenase IV (Worthington, USA), 0.5 mg/kg dispase (Worthington) and 1 μg/ml DNase (Sigma-Aldrich) in serum-free medium for 1 h at 37 °C. Fc receptors on isolated cells were blocked with normal mouse serum (1/4 dilution) and cells were stained with fluorochrome-conjugated mAbs specific for mouse CD45-PerCP-Cy5.5 (clone OX-1) and CD11b-APC-Cy7 (clone M1/70) (BD Biosciences), Ly6G-PE-Cy7 (clone 1A8), CD64-PE (clone X54-5/7.1), F4/80-BV421 (clone BM8) and CD31-BV605™ (clone 390) (Biolegend), MEFSK-4-APC (clone mEF-SK4) (Miltenyi Biotec) and the corresponding isotype controls. Cells were sorted on a fluorescence-activated cell sorting (FACS) Aria II (BD Biosciences) directly into RLY lysis buffer (Bioline) for mRNA expression analysis.

### Quantitative PCR

Quantitative PCR was performed as previously described [18, 28]. Briefly, total RNA was extracted from sorted joint cells using Isolate II RNA Mini Kit (Bioline) and reverse transcribed using Tetro Reverse Transcriptase (Bioline). Quantitative PCR (qPCR) was performed using the ABI Prism 7900HT sequence detection system (Applied Biosystems) and pre-developed TaqMan probe/primer combinations for murine *Col1a1, Ccl17, Ccr4, Mmp3, Mmp13* and *Ubc* (Life Technologies). Threshold cycle numbers were transformed to difference in cycle threshold (∆Ct) values, and the results expressed relative to the reference gene, *Ubc*.

### Statistics

Pain readings were analyzed using two-way analysis of variance (ANOVA). Histologic scores and osteophyte size were analyzed using one-way ANOVA or the Mann-Whitney two-sample rank test. Gene expression was analyzed using one-way ANOVA. Data are expressed as mean ± SEM; *p* ≤ 0.05 was considered statistically significant.

## Results

### Both IRF4 and CCL17 are required for CiOA pain and optimal disease development

We have shown that CiOA pain and disease development are GM-CSF dependent [8]. We have also reported that both GM-CSF-driven inflammatory pain and arthritis models (using exogenous GM-CSF) [17, 18], and GM-CSF-dependent inflammatory pain and arthritis models (e.g. zymosan-induced arthritis (ZIA)) are dependent on IRF4 and CCL17 [18], and on TNF [20]. Given these prior observations, we first determined whether arthritic pain and disease in the CiOA model are also dependent on TNF, IRF4 and/or CCL17.

**Fig. 1:**
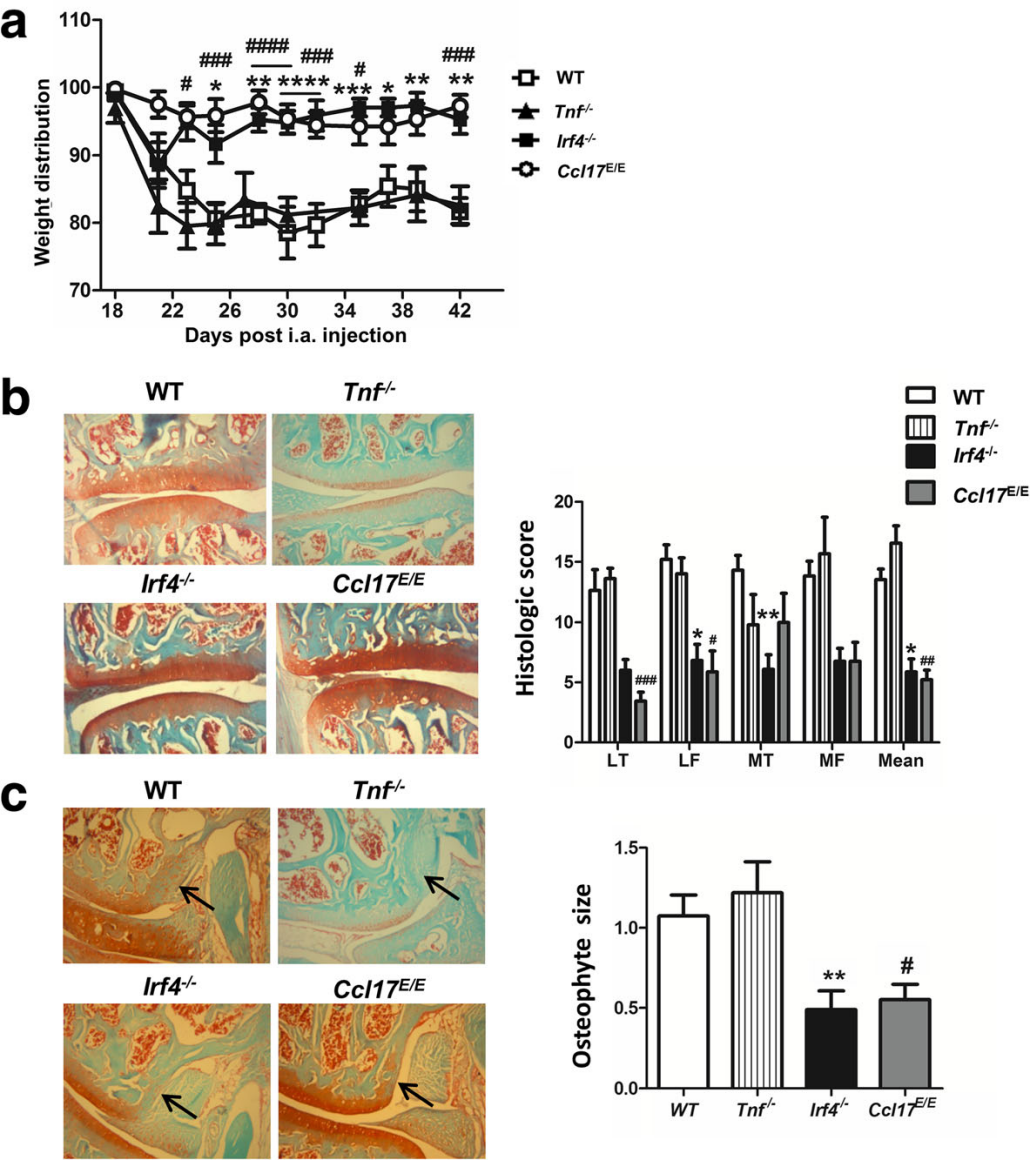
Interferon regulatory factor 4 (IRF4) and chemokine (c-c motif) ligand 17 (CCL17), but not TNF, are required for collagenase-induced osteoarthritis (CiOA) pain and optimal disease development. CiOA was induced in wild-type (WT), *Tnf*^*−/−*^, *Irf4*^−/−^ and *Ccl17*^E/E^ mice by intra-articular collagenase injection (see “Methods”). **a** Pain (change in weight distribution; see “Methods”) was monitored over time. **b** Representative histologic pictures of knee joints (Safranin O/fast green stain, original magnification ×100) and quantification of arthritis at day 42. **c** Representative histologic pictures of osteophytes (indicated by arrow) (Safranin O/fast green stain; original magnification ×100) and quantification of osteophyte size. Results are expressed as the mean ± SEM; n = 5–10 mice per strain (from two independent experiments). i.a., intra-articular; LT, lateral tibia; LF, lateral femur; MT, medial tibia; MF, medial femur. *p < 0.05, **p < 0.01, ***p < 0.001, ****p < 0.0001, WT vs. *Irf4*^−/−^ mice. ^#^*p* < 0.05, ^##^*p* < 0.01, ^###^*p* < 0.001, WT vs. *Ccl17*^*E/E*^ mice

CiOA was induced in *Tnf*^*−/−*^*, Irf4*^*−/−*^ and *Ccl17* gene-deficient (*Ccl17*^*E/E*^) mice and pain monitored over a 6-week period as previously described [8, 17, 18], by a change in weight distribution (using an incapacitance meter). As before [8], C57BL/6 WT mice developed pain around 3 weeks post collagenase injection; *Tnf*^*−/−*^ mice developed a similar degree of pain with similar kinetics, whereas neither *Irf4*^*−/−*^ nor *Ccl17*^*E/E*^ mice had any detectable pain (Fig. 1a). The collagenase-injected joints were evaluated histologically at 6 weeks (Fig. 1b) and disease was scored according to the published protocol [8]. While *Tnf*^*−/−*^ and WT mice had comparable scores, both *Irf4*^*−/−*^ and *Ccl17*^*E/E*^ mice had less arthritis compared to WT mice, with the mean score significantly lower for both strains compared to that observed in WT mice (Fig. 1b). Likewise, osteophyte size was similar in *Tnf*^*−/−*^ and WT mice, and was reduced in both *Irf4*^*−/−*^ and *Ccl17*^*E/E*^ mice (Fig. 1c).

**Fig. 2:**
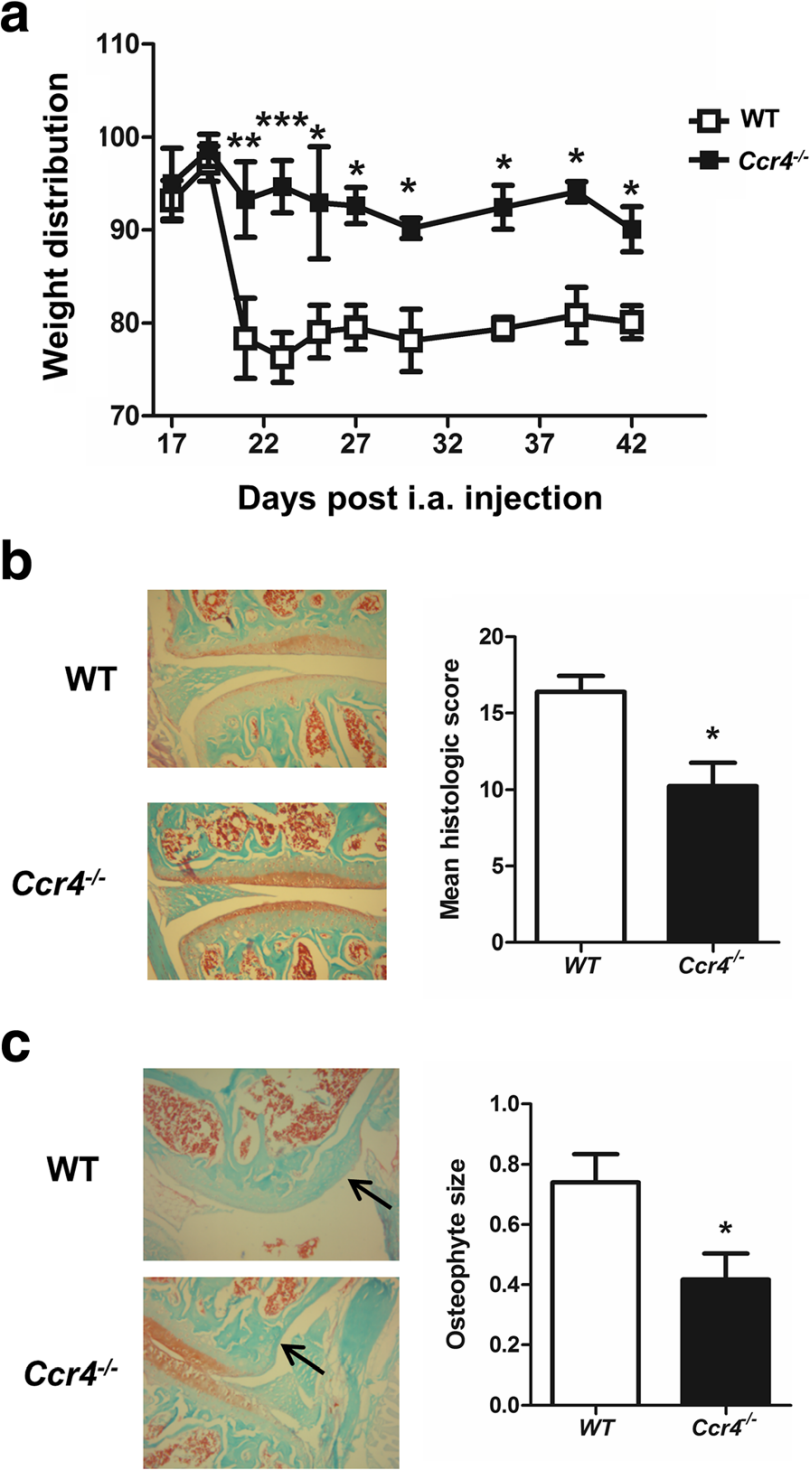
C-C motif chemokine receptor 4 (CCR4) is required for collagenase-induced osteoarthritis (CiOA) pain and optimal disease development. CiOA was induced in wild-type (WT) and *Ccr4*^*−/−*^ mice. **a** Pain (change in weight distribution; see “Methods”) was monitored over time. **b** Representative histologic pictures of knee joints (Safranin O/fast green stain, original magnification ×100) and quantification of arthritis at day 42. **c** Representative histologic pictures of osteophytes (indicated by arrow) (Safranin O/fast green stain; original magnification ×100) and quantification of osteophyte size. Results are expressed as the mean ± SEM; n = 5 mice per strain. i.a., intra-articular. *p < 0.05, **p < 0.01, ***p < 0.001, WT vs. *Ccr4*^−/−^ mice

Since C-C motif chemokine receptor 4 (CCR4) is usually considered to be the receptor for CCL17 [31], we also examined whether *Ccr4*^*−/−*^ mice are resistant to CiOA pain and disease development. *Ccr4*^*−/−*^ mice did not develop CiOA-induced arthritic pain (Fig. 2a) and also had significantly less overall disease and a reduction in osteophyte size (Fig. 2b and c, respectively) than WT mice.

The data above indicate that in addition to GM-CSF [8], both IRF4 and CCL17, the latter acting via CCR4, are required for CiOA pain and disease development and are consistent with our GM-CSF→IRF4→CCL17 pathway [18] being important in the CiOA model; however, TNF is not required.

### Therapeutic neutralization of CCL17 ameliorates CiOA pain and disease development

**Fig. 3:**
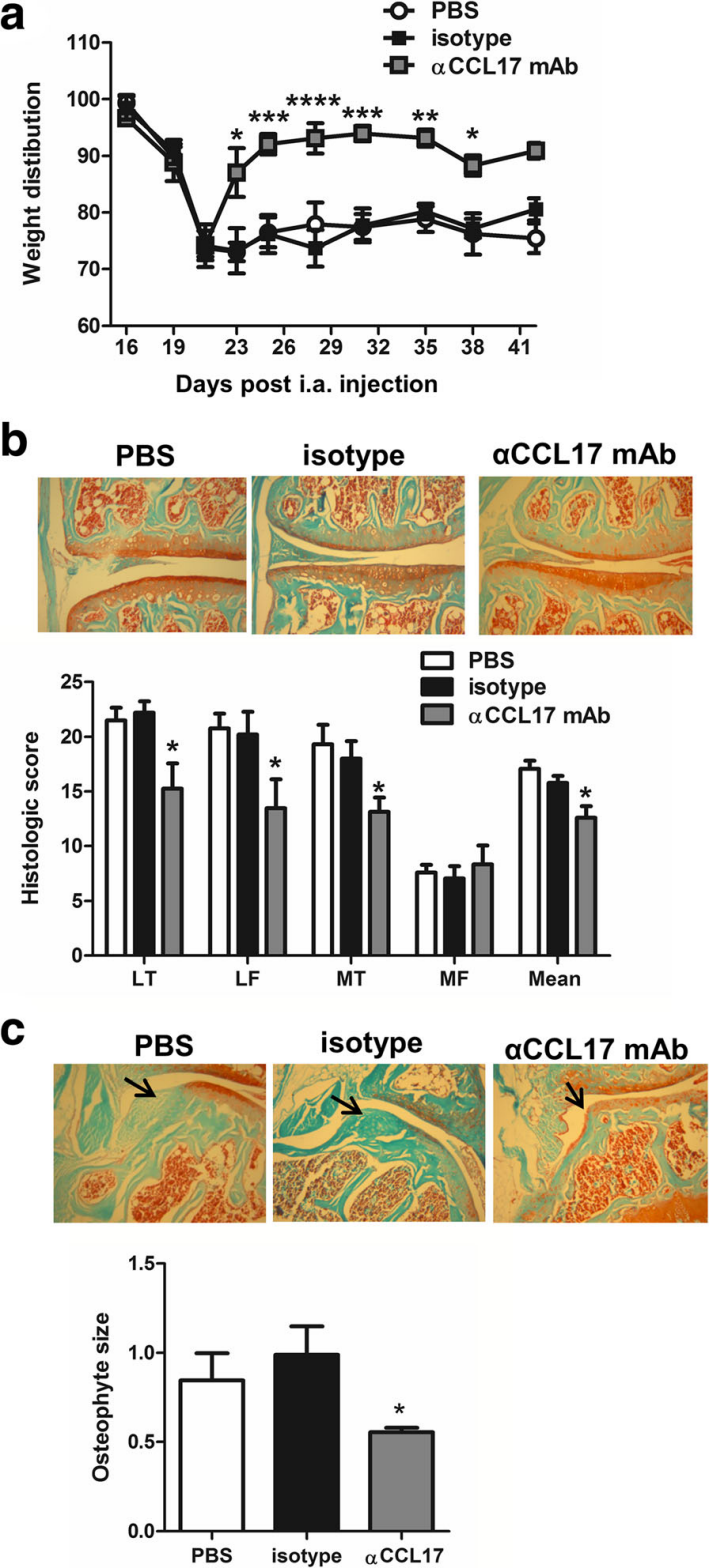
Therapeutic neutralization of chemokine (c-c motif) ligand 17 (CCL17) ameliorates collagenase-induced osteoarthritis (CiOA)-induced pain and arthritis*.* WT mice were induced with CiOA on day 0 and treated therapeutically from day 20 with PBS, isotype control or anti-CCL17 mAb twice weekly. **a** Change in weight distribution (pain) over time. **b** Representative histologic pictures of knee joints (Safranin O/fast green stain, original magnification ×100) and quantification of arthritis at day 42. **c** Representative histologic pictures of osteophytes (indicated by arrow) (Safranin O/fast green stain; original magnification ×100) and quantification of osteophyte size. Results are expressed as the mean ± SEM; n = 10 mice per strain (from two independent experiments). LT, lateral tibia; LF, lateral femur; MT, medial tibia; MF, medial femur; i.a., intra-articular. *p < 0.05, **p < 0.01, ***p < 0.001, ****p < 0.0001, isotype control vs. anti-CCL17

We evaluated next whether targeting CCL17 therapeutically, and therefore of potential clinical relevance, would suppress CiOA-induced pain and disease as we found previously for GM-CSF [8]. Following CiOA induction, once pain was evident (day 20), WT mice were treated with either PBS, isotype mAb or anti-CCL17 mAb until week 6. PBS-treated or isotype-treated mice continued to exhibit pain whereas pain was rapidly reversed in mice receiving anti-CCL17 mAb (Fig. 3a). This abolition of pain was maintained until week 6 (Fig. 3a). By histologic assessment, at 6 weeks the anti-CCL17 mAb-treated mice also had significantly milder disease and a reduction in osteophyte size compared to isotype-treated mice (Fig. 3b and c, respectively).

### Therapeutic inhibition of Jmjd3 ameliorates both CiOA pain and disease development

**Fig. 4:**
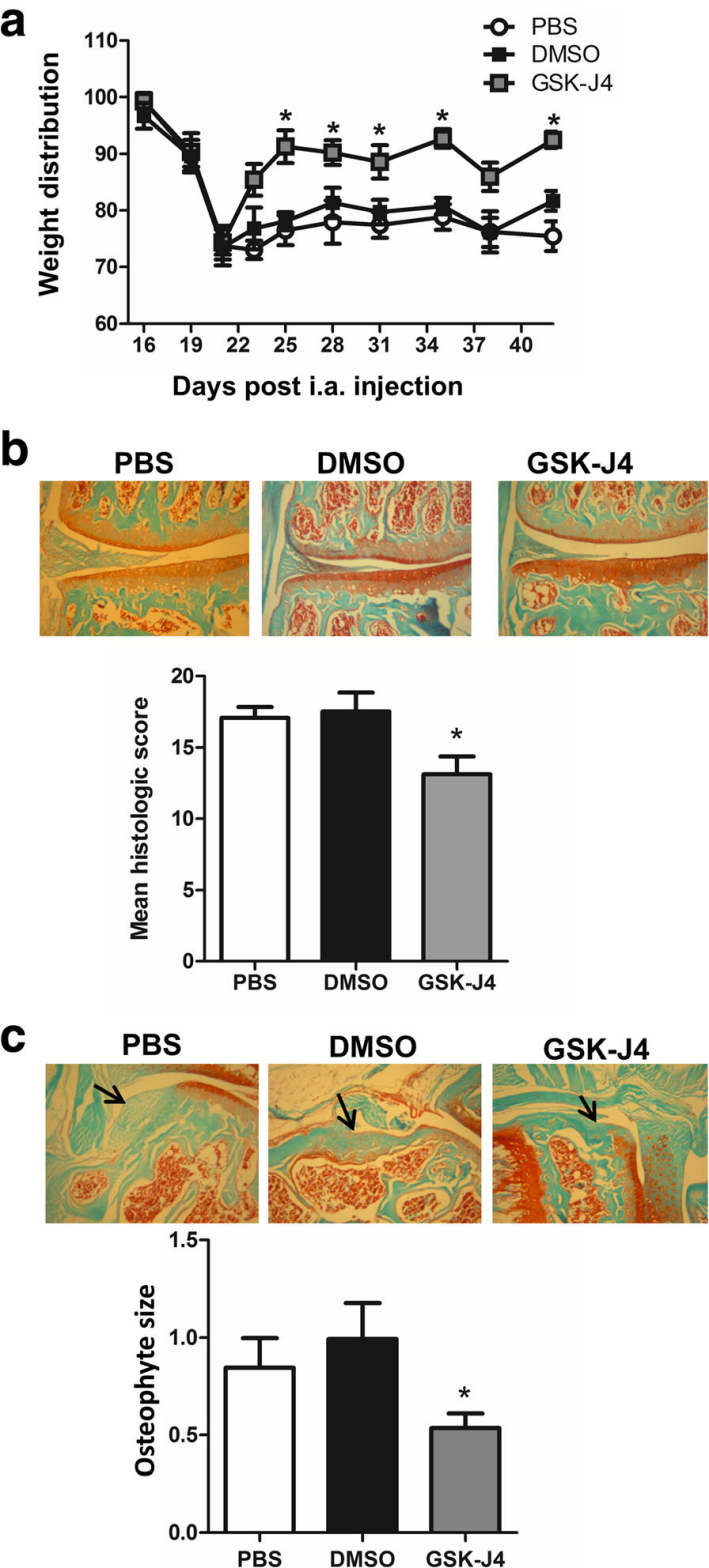
Therapeutic inhibition of Jmjd3 ameliorates both collagenase-induced osteoarthritis (CiOA) pain and disease development. Wild-type (WT) mice were induced with CiOA on day 0 and treated therapeutically from day 20 with PBS, dimethyl sulfoxide (DMSO) or GSK-J4 (0.5 mg/kg i.a.) for 5 consecutive days followed by twice weekly. **a** Change in weight distribution (pain) over time. **b** Representative histologic pictures of knee joints (Safranin O/fast green stain, original magnification ×100) and quantification of arthritis at day 42. **c** Representative histologic pictures of osteophytes (indicated by arrow) (Safranin O/fast green stain; original magnification ×100) and quantification of osteophyte size. Results are expressed as the mean ± SEM; n = 10 mice per strain (from two independent experiments). i.a., intra-articular. *p < 0.05, DMSO vs. GSK-J4

GM-CSF [8] and IRF4 (Fig. 1) are required for the development of CiOA arthritic pain and disease; also GM-CSF regulates IRF4 expression via Jmjd3 in human monocytes [18], *Irf4* being a direct target of Jmjd3-mediated demethylation [18, 32]. We thus examined whether the Jmjd3 inhibitor, GSK-J4 [18], could also reverse CiOA pain and disease. Following CiOA induction, when pain was evident (day 20), mice were treated with either PBS, vehicle (DMSO) or GSK-J4 until week 6. Treatment with PBS or DMSO had no effect on CiOA-induced pain; however, GSK-J4 treatment reversed it (Fig. 4a). Likewise, on histologic assessment arthritis development was lower and osteophyte size reduced in the GSK-J4-treated mice compared to vehicle-treated mice (Fig. 4b and c, respectively).

### Therapeutic inhibition of COX-2 ameliorates CiOA pain but not disease development

**Fig. 5:**
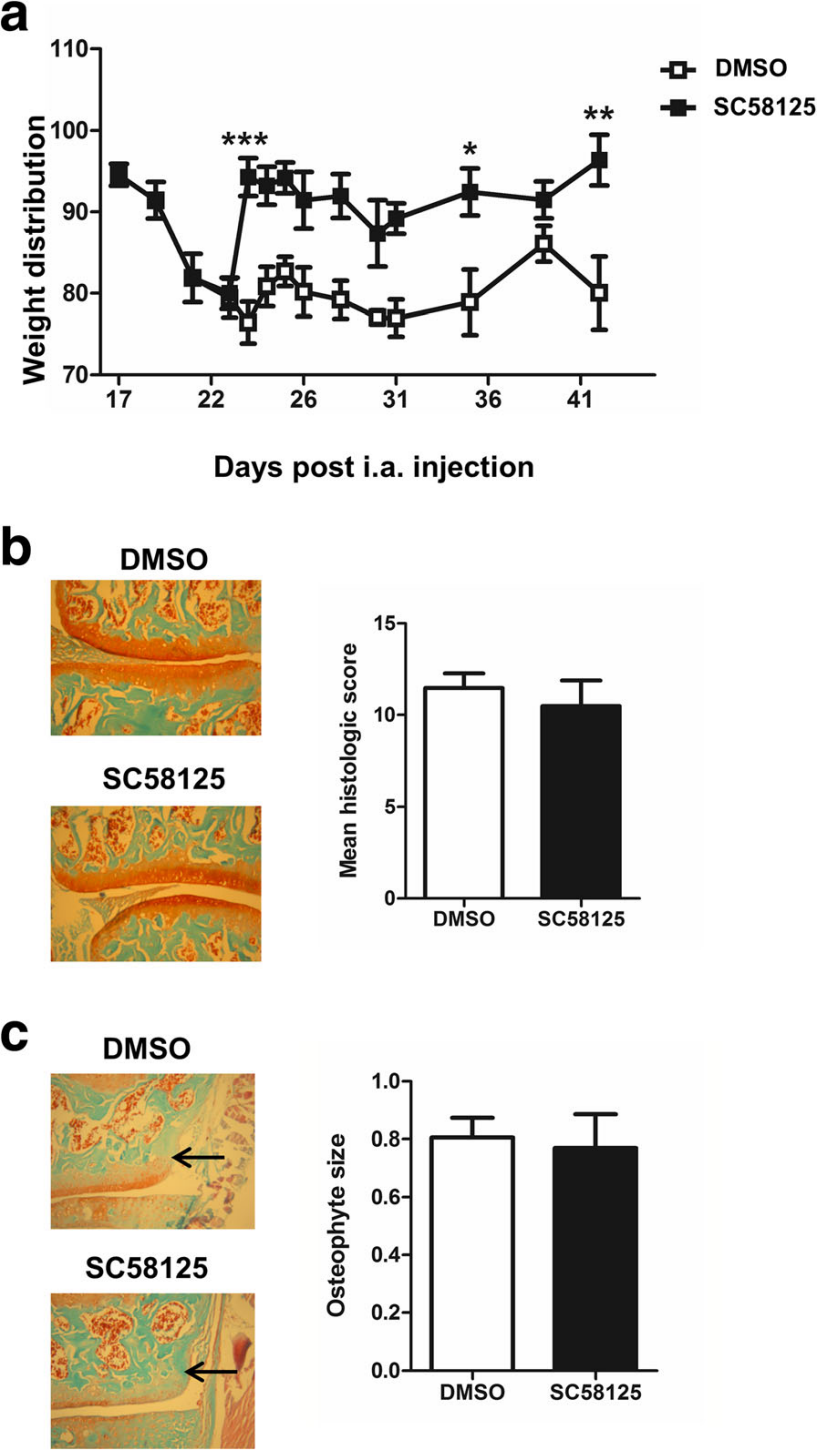
Therapeutic inhibition of cyclooxygenase 2 (COX-2) ameliorates collagenase-induced osteoarthritis (CiOA) pain, but not disease development. Wild-type (WT) mice were induced with CiOA on day 0 and treated therapeutically from day 23 with either dimethyl sulfoxide (DMSO) or the COX-2 specific inhibitor, SC58128 (1 mg/kg, i.p.) weekly. **a** Change in weight distribution (pain) over time. **b** Representative histologic pictures of knee joints (Safranin O/fast green stain, original magnification ×100) and quantification of arthritis at day 42. **c** Representative histologic pictures of osteophytes (indicated by arrow) (Safranin O/fast green stain; original magnification ×100) and quantification of osteophyte size. Results are expressed as the mean ± SEM; n = 5 mice per strain. i.a., intra-articular. *p < 0.05, **p < 0.01, ***p < 0.001, DMSO vs. SC58125

Given that inflammatory arthritis models requiring the GM-CSF→IRF4→CCL17 pathway were COX-dependent [18], we assessed whether CiOA was COX-2-dependent. When CiOA-induced pain was evident (day 23), mice were treated with the COX-2 inhibitor, SC58128, or vehicle (DMSO). SC58128 rapidly reversed the pain (Fig. 5a); however, by histology it had no effect on arthritis development or osteophyte size at 6 weeks (Fig. 5b and c, respectively).

### CCL17 is expressed in CiOA synovial macrophages and regulated by IRF4 and GM-CSF

**Fig. 6:**
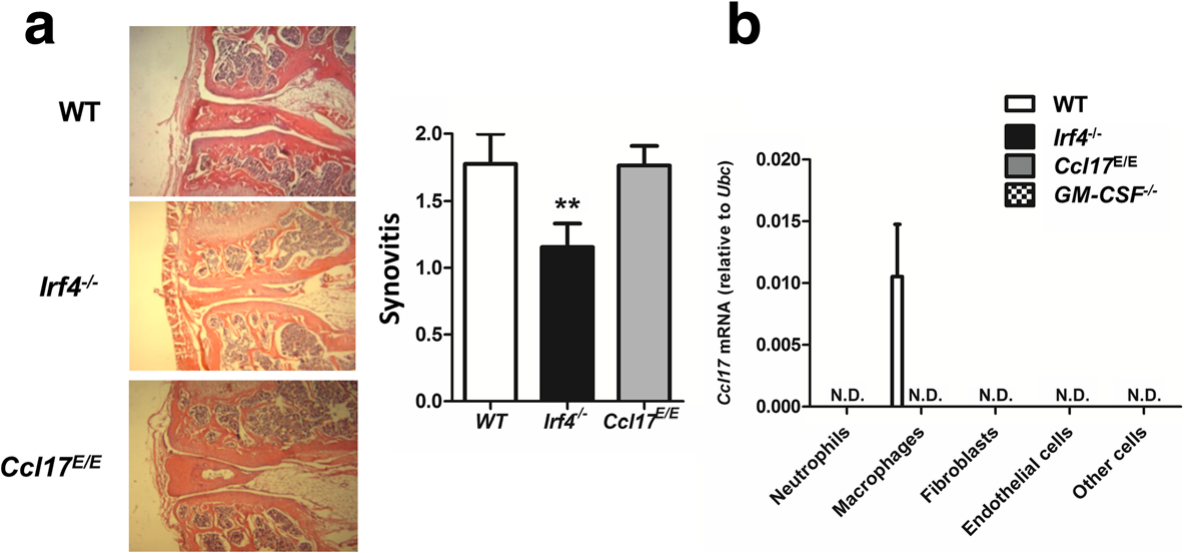
Chemokine (c-c motif) ligand 17 (CCL17) is expressed in collagenase-induced osteoarthritis (CiOA) synovial macrophages and regulated by interferon regulatory factor 4 (IRF4) and granulocyte macrophage-colony stimulating factor (GM-CSF). **a** CiOA was induced in wild-type (WT), *Irf4*^−/−^ and *Ccl17*^E/E^ mice and the joints were histologically assessed at week 2. Representative histologic pictures of knee joints (H&E stain, original magnification ×100) and quantification of synovitis; n = 5–10 mice per strain. **b** Synovial cell populations were sorted from joints at week 1 from WT, *Irf4*^*−/−*^, *Ccl17*^*E/E*^ and *GM-CSF*^*−/−*^ mice undergoing CiOA and *Ccl17* mRNA expression was measured; n = 3–6 mice per strain; Results are expressed as the mean ± SEM. N.D., not detected. **p < 0.01, WT vs. *Irf4*^−/−^ mice

Since synovitis in OA is often associated with greater symptoms such as pain and joint dysfunction and may promote more rapid cartilage degeneration [33] we explored a role for IRF4 and CCL17 in early synovitis in CiOA. The transient synovitis in CiOA was previously found to be GM-CSF dependent, being virtually absent in *GM-CSF*^*−/*−^ mice [8]. As expected, mild synovitis was observed in WT mice at 1 (data not shown) and 2 (Fig. 6a) weeks post CiOA induction [8]; interestingly, *Irf4*^*−/−*^ mice, but not *Ccl17*^*E/E*^ mice, had a slight reduction in synovitis (Fig. 6a).

In order to examine whether there was a difference in cell populations present during synovitis in the absence of IRF4 or CCL17, the synovial cell populations present at day 7 post CiOA induction from WT, *Irf4*^*−/−*^ and *Ccl17*^*E/E*^ mice were analysed by flow cytometry; *GM-CSF*^*−/−*^ mice were also included. Cells were identified as follows: CD45^+^ cells - neutrophils (CD11b^+^Ly6G^+^) and macrophages (CD11b^+^Ly6G^−^F4/80^+^CD64^+^); CD45^−^ cells - endothelial cells (CD31^+^mEF-SK4^−^), fibroblasts (CD31^−^mEF-SK4^+^) and other cells (CD31^−^mEF-SK4^−^) (Additional file 1 A) [34, 35]. The percentage of synovial neutrophils, but not of the other cell populations defined above, was reduced in the synovial cell populations from both *Irf4*^*−/−*^ and *GM-CSF*^*−/−*^ mice compared to WT mice, whereas no differences were seen in *Ccl17*^*E/E*^ mice compared to WT mice (Additional file 2).

The synovial cells were sorted to determine which populations were producing CCL17 at the mRNA level. To confirm CD45^−^CD31^−^mEF-SK4^+^ cells were fibroblasts, the fibroblast-associated *Col1a1* gene was found only in this population (Additional file 1 B). Consistent with our prior in vitro and in vivo data [18]), *Ccl17* mRNA was detected exclusively in macrophages from WT mice but was not present in the macrophages from *Irf4*^*−/−*^ or *GM-CSF*^*−/−*^ mice (Fig. 6b); *Ccr4* mRNA expression was widely expressed but predominantly in fibroblasts and endothelial cells (data not shown).

To determine whether the synovial cells from the different gene-deficient mice also had reduced mRNA expression for other genes compared to the synovial cells from WT mice, we examined certain genes implicated in the breakdown of extracellular matrices [36]. *Mmp3* (stromelysin-1) mRNA and *Mmp13* (collagenase-3) mRNA (Additional file 1 C and D, respectively) were exclusively expressed in fibroblasts, with lower expression seen in fibroblasts from *Irf4*^*−/−*^, *Ccl17*^*E/E*^ and *GM-CSF*^*−/−*^ mice compared to fibroblasts from WT mice.

These data indicate that while the degree of synovitis present early in CiOA in the absence of CCL17 is not affected, the gene expression of mediators potentially important for joint damage was. Furthermore CCL17 is produced by synovial macrophages in a GM-CSF-dependent and IRF4-dependent manner, consistent with our proposed pathway being active in these cells [18].

## Discussion

We have previously reported the importance of GM-CSF in the progression of pain and disease in the CiOA model [8]. Here we demonstrate that IRF4, CCL17 and CCR4 are also required. Therapeutic inhibition of CCL17 or Jmjd3 was successful in ameliorating the already established arthritic pain and disease. Thus our previously proposed GM-CSF→Jmjd3→IRF4→CCL17 pathway, first identified in human and murine monocytes/macrophages, appears to be important not only in the context of inflammatory arthritis and pain (e.g. ZIA) [18], but also in a model of OA, including in the development of the associated pain. However, TNF, which we have found before to be important for the initiation of ZIA pain and disease and mechanistically can use the same pathway leading to CCL17 formation via GM-CSF and JMJD3-regulated IRF4 formation [20], was not required for the development of CiOA pain or disease. CiOA pain, first detected at 3 weeks, was rapidly reversed by treatment with a specific COX-2 inhibitor but there was no effect on histologic scoring or osteophyte size.

CiOA shares some features with human OA, such as the development of synovitis, cartilage erosion and osteophytes [8, 11], which we have shown to be GM-CSF dependent [8]; there are a number of studies using this macrophage-dependent model in rodents (see, for example [8–13, 37, 38]). Interestingly, the proportion of synovial macrophages was not altered in the absence of GM-CSF, although there was a reduction in the proportion of neutrophils. A lack of IRF4 resulted in a slight reduction in early synovitis, with once again a reduction in the proportion of synovial neutrophils, while a lack of CCL17 had no effect; however, in the absence of either GM-CSF [8], IRF4 or CCL17 there was no pain development at week 3 and significantly reduced histologic scores and osteophyte size at week 6 (Fig. 1a-c). Thus, the degree of early synovitis observed upon deletion of IRF4 and CCL17, in contrast to that observed upon deletion of GM-CSF, does not correlate with the subsequent pain levels and histologic changes. In line with these observations, in OA the degrees of joint pain and structural change do not always overlap [5]. In the absence of IRF4 or CCL17, the activation states of the cells and the levels of associated inflammatory mediators present during synovitis may differ, which are likely important for the subsequent pathologic changes. In support of this mechanism, our gene expression analysis indicated that *Ccl17* mRNA is expressed in the CiOA synovial macrophages from WT mice, but not in those from *GM-CSF*^*−/−*^ and *IRF4*^*−/−*^ mice, data consistent with the involvement of the GM-CSF→IRF4→CCL17 pathway in the CiOA synovial macrophages, a cell type considered to be important in OA pathogenesis [2–6, 33, 39].

Importantly, therapeutic blockade of CCL17 ameliorated both CiOA pain and disease. CCL17 was originally considered to be a M2 cytokine due to its preferential attraction of T_H_2 lymphocytes [25, 40]. It can be produced by certain macrophage/dendritic cell populations [18, 23, 41, 42] and is elevated in many inflammatory conditions [18, 43–45] and in synovial fluid in OA [46]. CiOA synovitis in the absence of CCL17 suggests that CCL17 has other functions, apart from a chemotactic role [18]. In this connection, we have also observed in models of inflammatory arthritis, including lymphocyte-independent models, that a lack of CCL17 has more profound effects on cartilage damage and bone erosion than on cellular infiltration [18]. We have reported that systemic administration of CCL17 can drive arthritic pain in an inflamed joint in a COX-dependent manner [18]. Our data showing the therapeutic efficacy of a COX-2 inhibitor on CiOA pain (Fig. 5) is consistent with CCL17 being able to regulate joint eicosanoid levels in some manner. There are conflicting data as to whether the CCL17 receptor, CCR4, is expressed in neurons [47–50] as such expression would indicate the possibility of their direct activation by CCL17. Human microglial cells have been reported to express CCR4 [51], suggesting CCL17 could also act at this level in pain development. However, the blockade of CiOA pain by systemic anti-CCL17 mAb administration suggests a peripheral algesic action for CCL17, at least in this model.

The clinical syndrome of “OA” affects not only the composition, structure and function of articular cartilage but also the integrity of multiple joint tissues such as synovium, bone, etc., i.e. an appreciation has emerged that OA is a “whole joint” disease (see, for example, previous work [2–6, 33]). Also, as adult articular cartilage is avascular and aneural, pathogenic changes to non-cartilaginous joint tissues are of particular interest in understanding the source of pain generation in OA [33]. During OA progression, the synovial membrane is one source of proinflammatory and catabolic products, including matrix metalloproteinases (MMPs) and aggrecanases, which potentially contribute to articular matrix breakdown [33]. The synovial cell gene expression analysis described here indicated that *Ccr4* mRNA was expressed in a number of cell types, including fibroblasts, in the CiOA model while only fibroblasts appeared to express MMP3 and MMP13 which have been shown to be important for macrophage-mediated cartilage breakdown in experimental OA, including in CiOA [8, 52–54]. We found that, compared to WT mice, synovial fibroblasts from *GM-CSF*^*−/−*^, *Irf4*^−/−^ and *Ccl17*^*E/E*^ mice all had reduced mRNA expression of these MMPs and these gene-deficient mice also had reduced joint destruction and osteophyte size at 6 weeks post CiOA induction. As one possible mechanism, it could be that macrophage-derived CCL17 activates directly CCR4-expressing fibroblasts, which in turn augment MMP3 and MMP13 expression, leading to joint damage - both MMPs are expressed in synovial tissue from patients with early symptomatic OA [55]. Notably, CCL17-mediated CCR4 activation in other models is reported to up-regulate MMP13 [56, 57]. Importantly, cells in both cartilage and bone are also likely to express MMPs [58] and whether CCL17 acts directly on cartilage and bone requires further investigation, as does the relative contribution of the synovium, cartilage and bone to pain and joint destruction in this model.

Blockade of TNF has revolutionized RA treatment. We have recently shown that TNF can be linked to the GM-CSF→IRF4→CCL17 pathway, with the actions of TNF and GM-CSF being interdependent [20]. Interestingly, TNF is not required for CiOA pain and disease. It is not the only proinflammatory cytokine capable of associating with GM-CSF biology nor does GM-CSF-initiated inflammation necessarily involve TNF. For example, IL-1 can induce GM-CSF in a number of cell populations [59–61] and an IL-1-driven monoarticular arthritis model is GM-CSF dependent [62]. However, IL-1 is also not required for CiOA development [37]. How collagenase initiates the GM-CSF-dependent pathway in the CiOA model is unknown, as is the relationship of the pathway to other mediators important in this model, for example, the alarmins, S100A8 and S100A9 [38]. Consistent with our findings, anti-TNF treatment in the destabilization of the medial meniscus model did not ameliorate late-stage OA pain [63]. Some studies have assessed the benefit of anti-TNF therapies in patients with hand or knee OA with variable results [4, 6, 64, 65]. Better patient stratification and large, randomized, placebo-controlled trials would appear to be needed [4].

## Conclusions

From the findings described, we have evidence consistent with the GM-CSF→Jmjd3→IRF4→CCL17 pathway being important in CiOA, regulating both pain and disease. Whether the pathway is relevant to other experimental OA models is being explored. As for GM-CSF [8], therapeutic blockade of CCL17 was effective at ameliorating both pain and disease. As mentioned, low-grade inflammation is now being recognized as important in OA pathogenesis and progression [2–6, 33, 66] and pain has the highest impact on its burden [65]. Targeting GM-CSF or its receptor in RA is yielding promising results [15] and, as a result of prior findings in the CiOA model [8], a phase II trial in hand OA is currently underway [15]; however, given the possible adverse side effects associated with GM-CSF/GM-CSFR blockade, such as pulmonary alveolar proteinosis and infections [16, 67], targeting CCL17, which is elevated in the synovial fluid of patients with OA [46] and which we have found to be downstream of GM-CSF [18], may have some advantages for the treatment of not only inflammatory arthritis but also at least for some patients with OA.

## Additional files

Additional file 1: Synovial cell populations from joints at week 1 from WT, *Irf4*^*−/−*^, *Ccl17*^*E/E*^ and *GM-CSF*^*−/−*^ mice undergoing CiOA were sorted and gene expression measured. (A) Representative FACS plots showing synovial cell sorting strategy. CD45^+^ cells (II) were sorted into neutrophils (CD11b^+^Ly6G^+^) (III) and macrophages (CD11b^+^Ly6G^−^F4/80^+^CD64^+^) (IV); CD45^−^ cells (I) were sorted into endothelial cells (CD31^+^mEF-SK4^−^) (V), fibroblasts (CD31^−^mEF-SK4^+^) (VII) and other cells (CD31^−^mEF-SK4^−^) (VI). (B-D) mRNA expression in sorted synovial cell populations. (B) *Col1a1*, (C) *Mmp3* and (D) *Mmp13.* Results are expressed as the mean ± SEM; n = 3–6 mice per strain. N.D. not detected. *p < 0.05, **p < 0.01, WT vs. *Irf4*^−/−^, *Ccl17*^*E/E*^ or *GM-CSF*^−/−^mice. (PDF 257 kb)

Additional file 2: Proportions of synovial cell populations in knee joints from CiOA mice at day 7. (PDF 85 kb)

## Abbreviations

ANOVA: Analysis of variance
CCL17: Chemokine (c-c motif) ligand 17
CCR4: C-C motif chemokine receptor 4
CiOA: Collagenase-induced osteoarthritis
COX-2: Cyclooxygenase 2
DMSO: Dimethyl sulfoxide
Fc: Crystallisable fragment
GM-CSF: Granulocyte macrophage-colony stimulating factor
GM-CSFR: Granulocyte macrophage-colony stimulating factor receptor
IL: Interleukin
IRF4: Interferon regulatory factor 4
LF: Lateral femur
LT: Lateral tibia
mAb: Monoclonal antibody
MF: Medial femur
MMP: Matrix metalloproteinase
mRNA: Messenger RNA
MT: Medial tibia
OA: Osteoarthritis
PBS: Phosphate-buffered saline
RA: Rheumatoid arthritis
TNF: Tumour necrosis factor
WT: Wild-type
ZIA: Zymosan-induced arthritis
