# Supplementary figures and images for: CCL17 blockade as a therapy for osteoarthritis pain and disease
Source: Arthritis Res Ther. 2018 Apr 5;20:62. doi: 10.1186/s13075-018-1560-9 (PMC5887260; doi:10.1186/s13075-018-1560-9)

**A**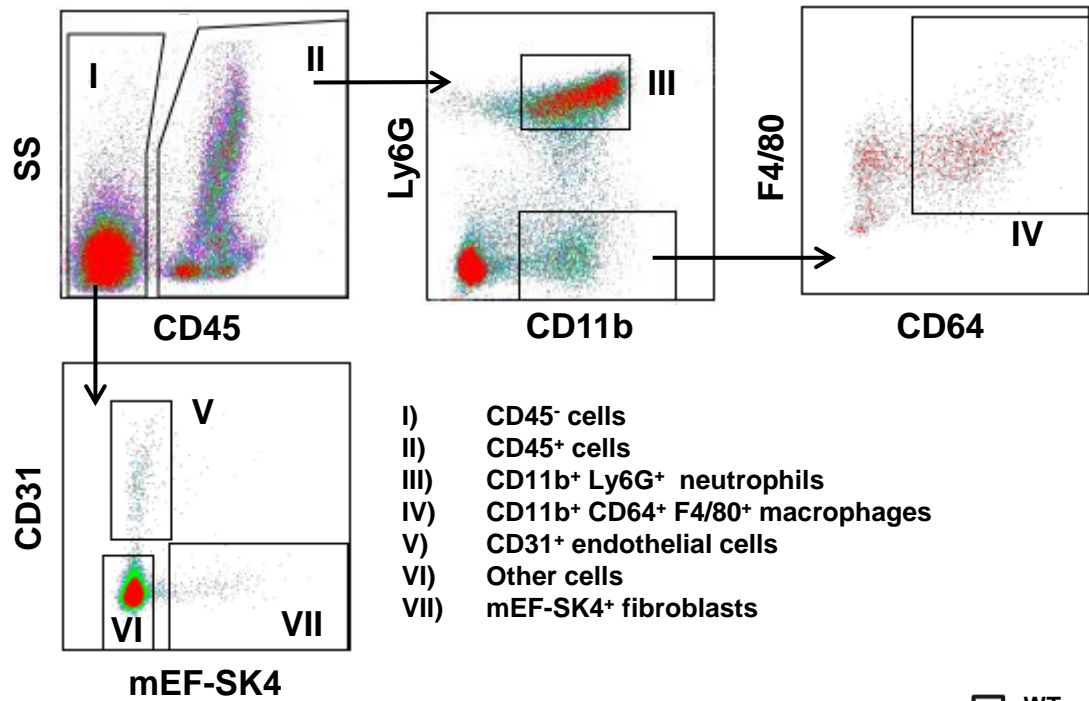**B**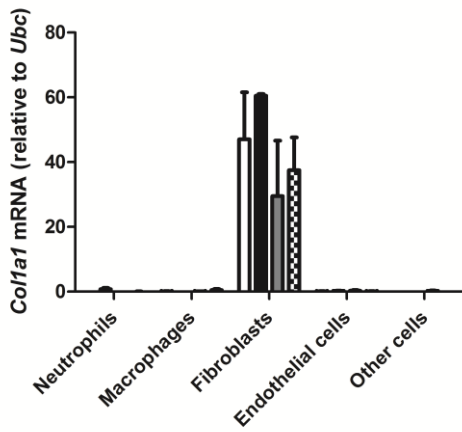**C**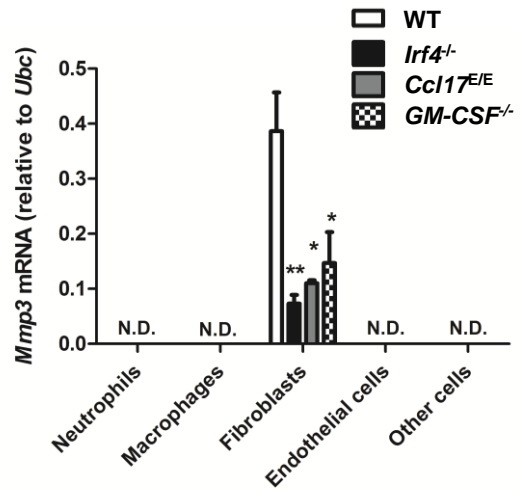**D**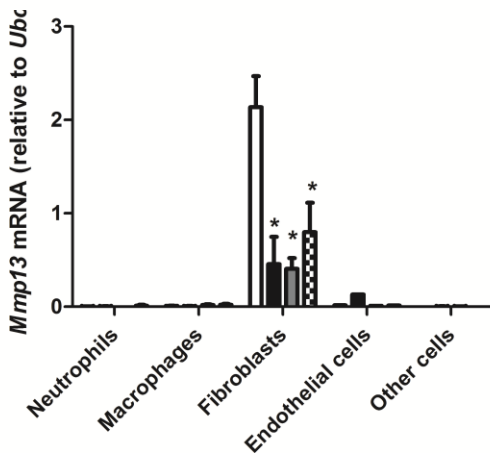

Supplement: Supplementary file 1 — Synovial cell populations from joints at week 1 from WT, Irf4−/−, Ccl17E/E and GM-CSF−/− mice undergoing CiOA were sorted and gene expression measured. (A) Representative FACS plots showing synovial cell sorting strategy. CD45+ cells (II) were sorted into neutrophils (CD11b+Ly6G+) (III) and macrophages (CD11b+Ly6G−F4/80+CD64+) (IV); CD45− cells (I) were sorted into endothelial cells (CD31+mEF-SK4−) (V), fibroblasts (CD31−mEF-SK4+) (VII) and other cells (CD31−mEF-SK4−) (VI). (B-D) mRNA expression in sorted synovial cell populations. (B) Col1a1, (C) Mmp3 and (D) Mmp13. Results are expressed as the mean ± SEM; n = 3–6 mice per strain. N.D. not detected. *p < 0.05, **p < 0.01, WT vs. Irf4−/−, Ccl17E/E or GM-CSF−/−mice. (PDF 257 kb) [file 13075_2018_1560_MOESM1_ESM.pdf]
