# Supplementary material for: CCL17 blockade as a therapy for osteoarthritis pain and disease
Source: Arthritis Res Ther. 2018 Apr 5;20:62. doi: 10.1186/s13075-018-1560-9 (PMC5887260; doi:10.1186/s13075-018-1560-9)
Supplement: Supplementary file 2 — Proportions of synovial cell populations in knee joints from CiOA mice at day 7. (PDF 85 kb) [file 13075_2018_1560_MOESM2_ESM.pdf]

Proportions of synovial cell populations in CiOA knee joints at day 7.

	WT	*Irf4*$^{-/-}$	*Ccl17*$^{E/E}$	*GM-CSF*$^{-/-}$
Neutrophils[1]	35.9 (2.5)	19.1 (1.4)*	42.7 (5.2)	22.8 (2.9)*
Macrophages[1]	6.4 (0.3)	7.8 (0.6)	9.8 (2.3)	8.0 (1.0)
Fibroblasts[2]	1.3 (0.2)	2.0 (0.2)	1.7 (0.2)	1.6 (0.4)
Endothelial cells[2]	2.6 (0.6)	3.1 (0.3)	3.4 (0.2)	3.1 (1.3)
Other cells[2]	95.7 (0.9)	94.3 (0.1)	94.1 (0.6)	94.9 (1.8)

Data expressed as percentage (SEM)

[1] Percentage of $CD45^+$ cells

[2] Percentage of $CD45^-$ cells

* $p<0.05$, WT vs. *Irf4*$^{-/-}$ or *GM-CSF*$^{-/-}$ mice
